# TRAF6 promotes spinal microglial M1 polarization to aggravate neuropathic pain by activating the c-JUN/NF-kB signaling pathway

**DOI:** 10.1007/s10565-024-09900-6

**Published:** 2024-07-12

**Authors:** Yu Zhao, Tiegang Li, Lichun Zhang, Jun Yang, Feng Zhao, Yu Wang, Yi Ouyang, Jiahui Liu

**Affiliations:** 1 Department of Emergency, Shengjing Hospital of China Medical University, Shenyang, Liaoning Province 110136 People’s Republic of China; 2 Department of Neurology, the First Hospital of China Medical University, No. 155 Nanjing North Street, Heping District, Shenyang, Liaoning Province 110001 People’s Republic of China

**Keywords:** Neuropathic pain, TRAF6, Microglia polarization, c-JUN/NF-kB pathway

## Abstract

**Background:**

The neuropathic pain with complex networks of neuroinflammatory activation severely limits clinical therapeutic research. TNF receptor-associated factor 6 (TRAF6) is associated with multiple inflammatory diseases. However, there remains confusion about the effects and mechanisms of TRAF6 in neuropathic pain.

**Methods:**

A chronic constriction injury (CCI) model was developed to simulate neuralgia *in vivo*. We overexpressed or knocked down TRAF6 in CCI mice, respectively. Activation of microglia by TRAF6, the inflammatory response, and disease progression were inspected using WB, qRT-PCR, immunofluorescence, flow cytometry, and ELISA assays. Moreover, the mechanism of M1/M2 polarization activation of microglia by TRAF6 was elaborated in BV-2 cells.

**Results:**

TRAF6 was enhanced in the spinal neurons and microglia of the CCI mice model compared with the sham operation group.. Down-regulation of TRAF6 rescued the expression of Iba-1. In response to mechanical and thermal stimulation, PWT and PWL were improved after the knockdown of TRAF6. Decreased levels of pro-inflammatory factors were observed in TRAF6 knockdown groups. Meanwhile, increased microglial M1 markers induced by CCI were limited in mice with TRAF6 knockdown. In addition, TRAF6 overexpression has the precise opposite effect on CCI mice or microglia polarization. We also identifed that TRAF6 activated the c-JUN/NF-kB pathway signaling; the inhibitor of c-JUN/NF-kB could effectively alleviate the neuropathic pain induced by upregulated TRAF6 in the CCI mice model.

**Conclusion:**

In summary, this study indicated that TRAF6 was concerned with neuropathic pain, and targeting the TRAF6/c-JUN/NF-kB pathway may be a prospective target for treating neuropathic pain.

**Graphical Abstract:**

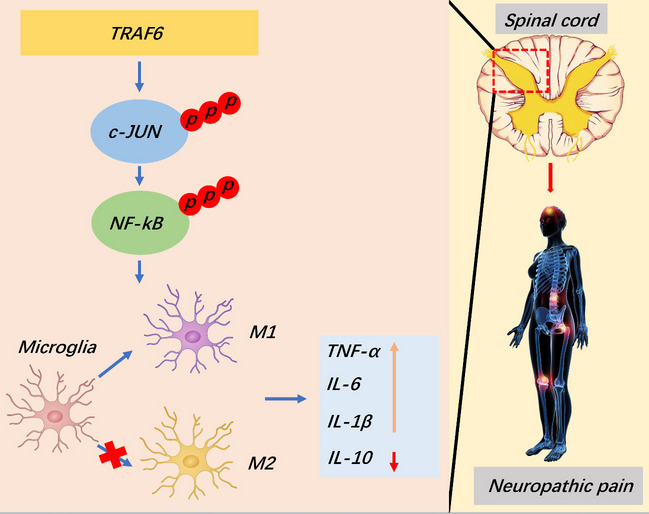

**Supplementary Information:**

The online version contains supplementary material available at 10.1007/s10565-024-09900-6.

## Introduction

Neuropathic pain is a chronic painful condition that cannot be effectively controlled due to complex etiologies (Zhao et al. 2018a; b). The clinical features of the pain, including spontaneous pain, hyperalgesia, and paresthesia, result in a decreased quality of the patient's life (Zhao et al. 2018a; Descalzi et al. 2015). Lesion or dysfunction induces the release of neurotransmitters, neurotrophic factors, cytokines, and chemokines (Wang et al. 2018). By lowering the activation threshold of olfactory signals in the periphery, the substances increase the sensitivity and excitability of primary sensory neurons, causing peripheral sensitization (Wang et al. 2018; Iyengar et al. 2017; Silva et al. 2017). Although researchers have shown that nociceptors and modulators are involved in neuropathic pain, the mechanisms of neuropathic pain remain poorly understood (Wang et al. 2018).

Microglia are innate immune and principal phagocytes of the brain, serving as protective guardians of the central nervous system (CNS). In neuropathic pain cases, the M1/M2 phenotypic homeostasis among pro-inflammatory and anti-inflammatory mediators is destroyed and skewed in favor of M1 macrophages. The sustained generation of M1 proinflammatory molecules contributes to neuroinflammation and long-term neuropathic pain development. In contrast, M2 activation outcomes in suppression of inflammatory cell recruitment, phagocytosis of cellular debris, and restoration of internal environmental homeostasis. A series of signaling factors that regulate microglia polarization-mediated inflammatory response and alleviate neuropathic pain and neuroinflammation were discerned, including Orai1 channel (Tsujikawa et al. 2023), Dickkopf (DKK) 3 (Zhang et al. 2022), TRPV4 (Hu et al. 2023), and so on. Upon these findings, investigations have also established a range of compounds that inhibit CCI-induced NP by promoting microglial cell M2 polarization, such as salvinorin (Li et al. 2023), botulinum toxin (Gui et al. 2020b), etc. These studies indicate that modulators that switch microglia from an M1 to M2 phenotype are a prospective therapy for managing neuropathic pain.

TRAF6 is instrumental for immunological effector cell function and is an inflammatory signaling factor (O'Neill et al. 2021). Studies are continuously validating that TRAF6, beyond mediating tumor T-cell immunity (Yu et al. 2023), participates in activating microglial inflammatory responses in neurodegenerative diseases such as cerebral hemorrhage secondary to brain injury and Parkinson's (Han et al. 2020; Yang et al. 2020), playing an essential role in this regard. It was pointed out that TRAF6 can activate NF-κB, MAPK, and Wnt/B-catenin pathways by interacting with protein. However, the mechanism of neuropathic pain has not yet been clarified. TRAF6 has been appreciated for its potential in neuropathic pain in recent years. Others have observed in chronic inflammatory hypersensitivity that direct or indirect inhibition of TRAF6 reduces pain hypersensitivity due to microglia activation in the spinal dorsal horn (Hua et al. 2022; Lu et al. 2022).

This study was designed to characterize the effects of TRAF6 on neuropathic pain. We simulated neuropathic pain with a CCI mouse model and assessed the expression and localization of TRAF6 in spinal cord neuronal cells, microglia, and astrocytes in CCI mice. Functional experiments confirmed the effects of TRAF6 knockdown on pain sensitivity, spinal cord injury, and inflammatory responses. Meanwhile, the modulatory impact of TRAF6 on microglia M1/M2 polarization state and its underlying molecular mechanisms were dually proven *in vivo* and *in vitro*. Moreover, the findings demonstrated a potential biomarker role for TRAF6 in neuropathic pain.

## Materials and methods

### Animals

Male C57BL/6 (Medical Laboratory Animal Center, Guangzhou, China) mice (6–8 weeks old, 24–30 g) were housed in a temperature/humidity environment of 24 °C/50–60%, with normal ventilation and light/dark cycles (12 h), and fed and watered ad libitum. Animal experiments were ethically licensed by the Ethics Committee (Shengjing Hospital, China Medical University) and conducted in accordance with the guidelines.

### Mouse CCI model establishment

Neuralgia pain model was simulated using CCI. CCI model was induced as described previously (Wang et al. 2020). In Brief, mice with anesthetized (0.8% pentobarbital sodium, ip), incisions were made to expose the left sciatic nerve, and loose ligations (1-mm spacing) were applied to the trunk nerves at the branches using ligature wires. The muscles of the ipsilateral hind limb were observed to show slight twitching. The sham-operated group was operated but not ligated.

### AAV injection and experimental design

To verify the impact of TRAF6 on CCI, adeno-associated virus (AAV) was injected intrathecally using a glass micropipette (Hamilton). The dosages of AAV-shRNA or AAV-TRAF6 were chosen from previous reports (He et al. 2020). Specifically, AAV (10 μl) was injected 2 weeks before CCI. AAV-TRAF6 (5.0 × 10 ^12^ vg/ml) vector and its control vector AAV-NC (5.0 × 10 ^12^ vg/ml), AAV-shRNA-TRAF6 (1.5 × 10 ^12^ vg/ml) and its control vector AAV-shRNA (1.6 × 10 ^12^ vg/ml) were purchased from Viligo Biotechnology Co. Ltd (Hangzhou, China). c-JUN inhibitor SP600125 (15 mg/kg, i.p, MCE, USA) was injected on postoperative days 4–8. Thermal hyperalgesia and mechanical allodynia were performed using the CCI model (3, 5, 7, 14, and 21 days). To confirm the quality of transfection, we provided the results of AAV fluorescence identification (Figure S1).

### Behavioral tests

The paw withdrawal threshold (PWT) was calculated using the thin filament stimulation method (Von Frey, 0.07 and 0.4 g, Stoelting Co.), as mentioned previously (He et al. 2020). For paw withdrawal latency (PWL), thermal testing was evaluated using IITC Inc./Life Science Instruments (Model 336, Science Instruments, Woodland Hills, CA). After behavioral tests, the spinal cord was saved for subsequent detection.

### Cell culture and transfection

Ventilated BV2 microglia were cultured in DMEM medium (10% fetal bovine serum, 37 °C, 5% CO2). Lipopolysaccharide (LPS) (10, 100, 1000 ng/ml, Sigma-Aldrich) was then applied to BV2 cells for 24 h to imitate the activation of BV2 microglia.

In accordance with the instructions, H293T cells were transfected with AAV-shRNA, AAV-shNC, AAV-NC, or AAV-TRAF6 for 48 h. The TRAF6 or c-JUN recombinant AAV viral solution was incubated with BV-2 cells, and subsequent experiments were performed. BV-2 Cells (1 × 10^5^ cells/well) were transfected with 40 nM TRAF6, sh-TRAF6, sh-c-JUN, or negative shRNA. After 24 h, transfection efficiency was assessed using WB.

### Flow cytometry

To study microglia polarization, spinal cord tissue was removed and cut up, prepared into single cell suspensions by digestion with 0.25% trypsin (Invitrogen), and incubated with CD86-FITC (555018, BD Biosciences, 1:200) and CD206-APC (550889, BD Biosciences, 1:200) for 30 min in the dark on ice. Analysis using flow cytometry (FACSVerse 8, BD) with visualization (FlowJo software version 7.6.1).

### Quantitative real-time PCR

Total spinal cord RNA was extracted (Trizol, Thermofisher, USA), and then processed (SuperScript IV, Thermofisher, USA) for cDNA using a reverse transcription kit. Table S1 lists measurements, markers, and associated PCR primers employed in.this study.

### Western blot

Total proteins(RIPA, Beyotime, China) were extracted for quantification, followed by electrophoresis on a gel (SDS-10% polyacrylamide). Subsequently transferred to PVDF membranes for containment (Tris, 3% skimmed milk, 1 h), primary antibodies were incubated overnight: anti-TRAF6 (A0973, ABclonal, 1:1000), anti-iNOS (ab178945, Abcam,1:1000), anti-Arg1 (ab96183, Abcam, 1:1000), anti-c-JUN (SC-74543, Santa Cruz, 1:10000), anti-NF-kB p65 (3034, Cell Signaling, 1:1000), anti-c-JUN (phosphor S70) (ab30620, Abcam, 1:1000), anti-NF-kB p65 (phosphor S536) (3033, Cell Signaling, 1:1000), anti-GAPDH (60008–1-Ig, Proteintech, 1:1000).

Washed in PBS, the secondary antibody (ab288151, Abcam, 1:3000) was incubated (37 °C, 1 h), followed by visualization and quantitative analysis.

### Immunofluorescence assay

Fixed spinal cord tissues were paraffin-embedded, deparaffinized and repaired with antigens (boiled, 10 min), blocked in goat serum (room temperature, Solarbio,15 min), and incubated with primary antibodies (4 °C, 24 h): anti-TRAF6 (A0973, ABclonal, 1:1000), anti-iNOS (Abcam, ab178945, 1:500), anti-Arg1 (Abcam, ab96183, 1:500), anti-Iba-1 (Abcam, ab178846, 1:500), Neuronal Marker anti-NeuN (Abcam, ab177487, 1:1000), anti-GFAP (Proteintech, 16825–1-AP, 1:50). After PBS washing, the secondary antibody (Alexa Fluor 488/Alexa Fluor 546, Beyotime, China, 1:200) was reacted for 90 min, and observed under a fluorescence microscope.

### ELISA assay

The IL-1β, TNF-α, IL-6, and IL-10 levels in the spinal cord were measured using Elisa kits (Beyotime). Quantification was performed using a microplate reader (Molecular Devices, USA, OD 450 nm). Samples concentrations were counted from the prepared standard curve.

### Statistical analysis

Data were visualized and analyzed (means ± SD) using GraphPad Prism 7 (GraphPad Software, Inc.). Data were statically analyzed for differences using a t-test or one-way or two-way analysis of variance (ANOVA), with *p* < 0.05 or* p* < 0.01 considered significantly different.

## Results

### TRAF6 was up-regulated in CCI model

**Fig. 1:**
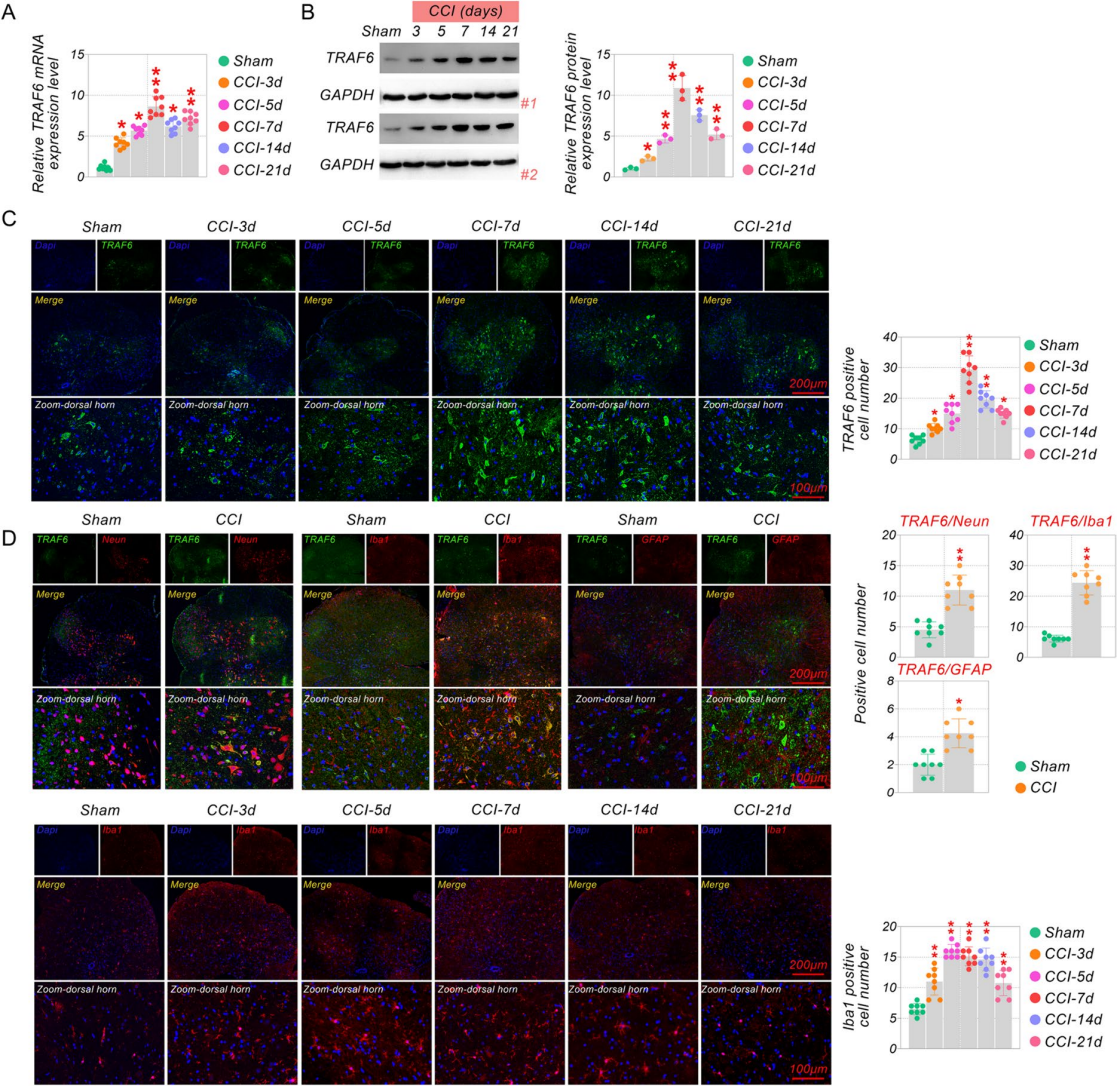
TRAF6 was up-regulated in mice CCI model. (**A**) QRT-PCR analyzed the mRNA expression of TRAF6 on different days in the sham or CCI group. (**B**) TRAF6 levels in the spinal cord of different days in the sham or CCI groups were detected by Western blotting. (**C**) Representative immunofluorescence images of TRAF6 on different days. (**D**) Immunofluorescence staining was tested for the co-expression of TRAF6, NeuN, Iba-1, and GAFP in the spinal cord of CCI mice with TRAF6. (**E**) Representative immunofluorescence images of Iba-1 on different days. *p < 0.05, **p < 0.01 vs Sham. Scale bar: 200/100 μm. #1 and #2 represent repeated trials

To simulate neuropathic pain, sciatic nerve ligation surgery was used for establishing the CCI mice model. Compared with the sham group, the qRT-PCR, and western blot analysis showed that the TRAF6 level was rapidly and significantly elevated in CCI mice (Fig. 1 A-B). The enhancement of TRAF6 started from day 3, reached a peak on day 7, and maintained until day 21. Immunofluorescence assays reconfirmed this result (Fig. 1C). Then, we observed the co-expression and distribution of TRAF6 with neuronal marker NeuN, microglia marker Iba-1, and astrocyte marker GFAP in sham and CCI mice as CCI progressed using immunofluorescence staining (Fig. 1D). Co-expression of TRAF6 with NeuN, Iba-1, and GFAP was increased in the CCI model, and the variation was more pronounced in microglia. Therefore, we re-examined the tendency of Iba-1 changes under different days of CCI. The results revealed that Iba-1 peaked on the third day and lasted until day 14, when it began to decline and was lowest on day 21 (Fig. 1E). Our results implied that the sustained activation state of microglial might be positively correlated with TRAF6 expression. When TRAF6 expression began to decline, the microglial activation state also began to reduce. However, it did not precisely meet the same time change pattern in CCI-induced neuropathic injury.

### Inhibition of TRAF6 relieved hyperalgesia induced by CCI

**Fig. 2:**
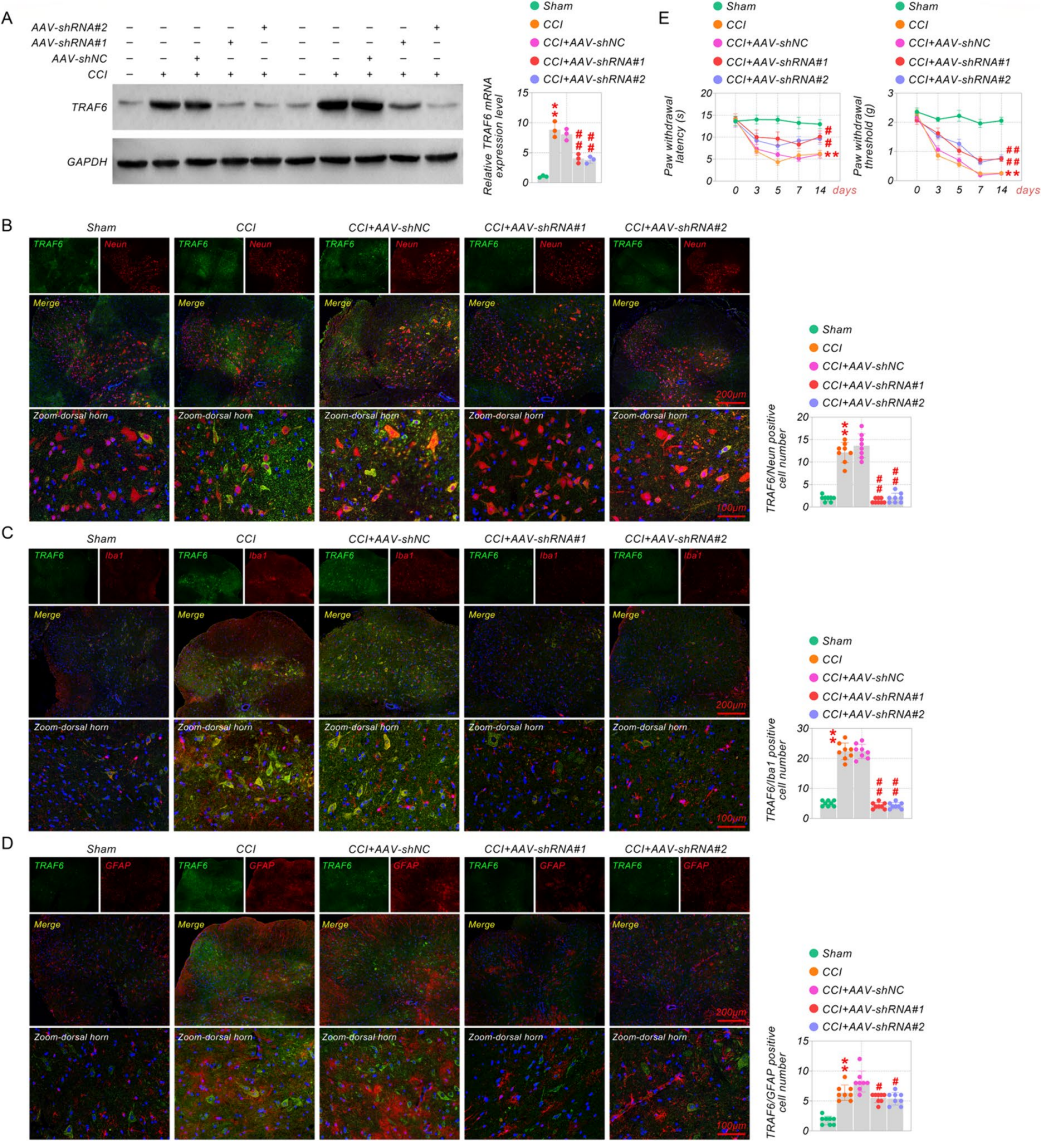
Inhibition of TRAF6 relieved hyperalgesia induced by CCI. (**A**) The western blot and qRT-PCR assay tested AAV-shRNA or AAV-shNC transfection efficiency in CCI-induced mice. (**B-D**) The immunofluorescence was further employed to detect co-expression of TRAF6 with NeuN, GFAP, and Iba-1, respectively, in CCI-induced mice after intrathecally injecting AAV-shRNA or AAV-shNC. (**E**) Mechanical allodynia was estimated by Von Frey filaments detection of paw withdrawal thresholds, and thermal hyperalgesia was calculated by radiant heat detection of paw withdrawal latency. **p < 0.01 vs Sham, and ^#^*p* < 0.05, ^##^*p* < 0.01 vs CCI. Scale bar: 200/100 μm

To characterize the underlying effect of TRAF6 in neuropathic pain, we knocked down TRAF6 by intrathecal injection using short hairpin RNA (shRNA) of adeno-associated virus (AAV). Western blot assay showed that AAV-shRNA#1 and AAV-shRNA#2 effectually declined the expression of TRAF6 compared with the AAV-shNC group (Fig. 2A). Furthermore, co-immunofluorescence staining demonstrated that TRAF6 knockdown (AAV-shRNA) reduced the co-expression of TRAF6 in neuronal, microglia, and astrocytes (Fig. 2B-D). We performed mechanical and thermal tests to determine the effect of TRAF6 inhibition on motor ability and pain sensitivity in CCI mice. As shown in Fig. 2E, the PWT and PWL were reduced by CCI compared with the sham group; TRAF6 knockdown significantly alleviated these symptoms. The results suggest that inhibition of TRAF6 alleviates CCI-induced neuropathic pain sensitivity.

### Inhibition of TRAF6 modulated CCI-induced microglia polarization to alleviate CCI-induced inflammatory reaction

**Fig. 3:**
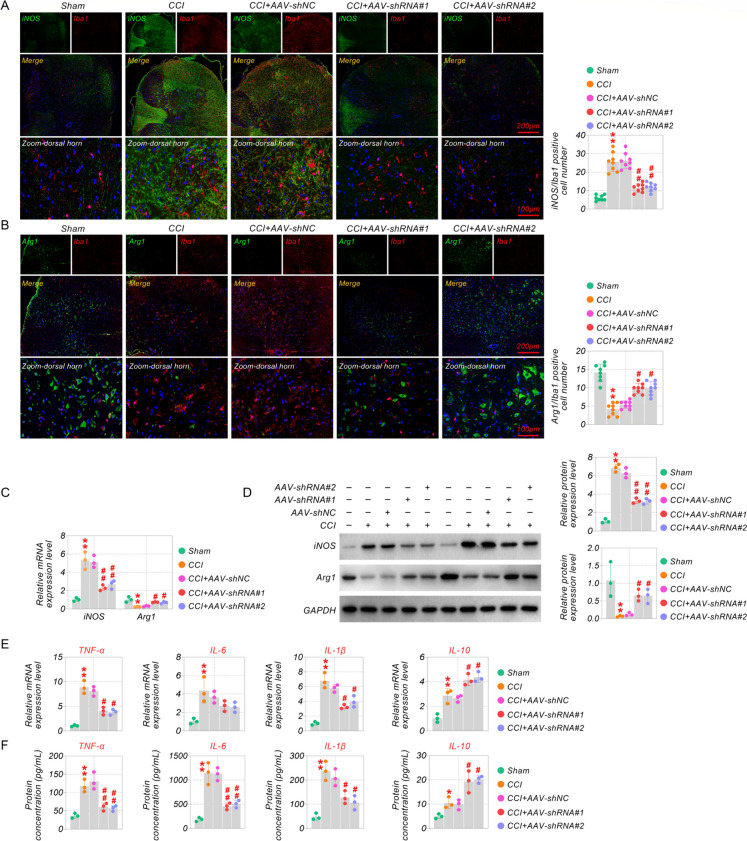
Inhibition of TRAF6 modulated CCI-induced microglia polarization to alleviate CCI-induced inflammatory reaction. (**A-B**) Co-expression levels of Iba-1 with iNOS and Arg1, respectively, with or without AAV-shRNA were examined using co-immunofluorescence staining in CCI-induced mice. (**C**) QRT-PCR and (**D**)Western blot assay were utilized to test the iNOS and Arg1 levels. (**E**) QRT-PCR and (**F**) Elisa assay kits were used to test the release of inflammatory cytokines. *p < 0.05, **p < 0.01 vs Sham, and ^#^*p* < 0.05, ^##^*p* < 0.01 vs CCI. Scale bar: 200/100 μm

Microglial activation has a vital function in neuropathic pain. M1 phenotype of microglia is usually dominant in neuropathic pain (Gui et al. 2020a). To examine the effect of TRAF6 on microglia activation in CCI-induced mice, we evaluated M1-labeled iNOS and M2-labeled Arg1 levels in microglia. The immunofluorescence staining displayed that TRAF6 knockdown reversed the enhanced iNOS expression and reduced Arg1 levels compared with the AAV-shNC group. The immunofluorescence staining revealed that Iba-1 was correlated positively with iNOS and negatively with Arg1 (Fig. 3A-B). Meanwhile, the qRT-PCR and western blot assay results proved that TRAF6 knockdown could decrease iNOS expression and increase Arg1 expression (Fig. 3C and D). This test exhibited that TRAF6 suppression could depress CCI-induced M1 polarization of microglia and encourage the anti-inflammatory M2 phenotype.

Inflammation is a common and latent mechanism in neuropathic pain. Then, we determined the level of inflammatory cytokines using qRT-PCR and Elisa. As shown in Fig. 3E and F, inflammatory cytokines were substantially more abundant in the CCI group compared with the sham group. Nonetheless, the elevated contents of pro-inflammatory cytokines IL-1β, IL-6, and TNF-α levels were depressed by TRAF6 knockdown, and IL-10 (anti-inflammatory) was promoted. The data reveals that inhibition of TRAF6 alleviates excessive inflammation induced by CCI operation.

### TRAF6 promoted polarization of microglia M1 phenotype

**Fig. 4:**
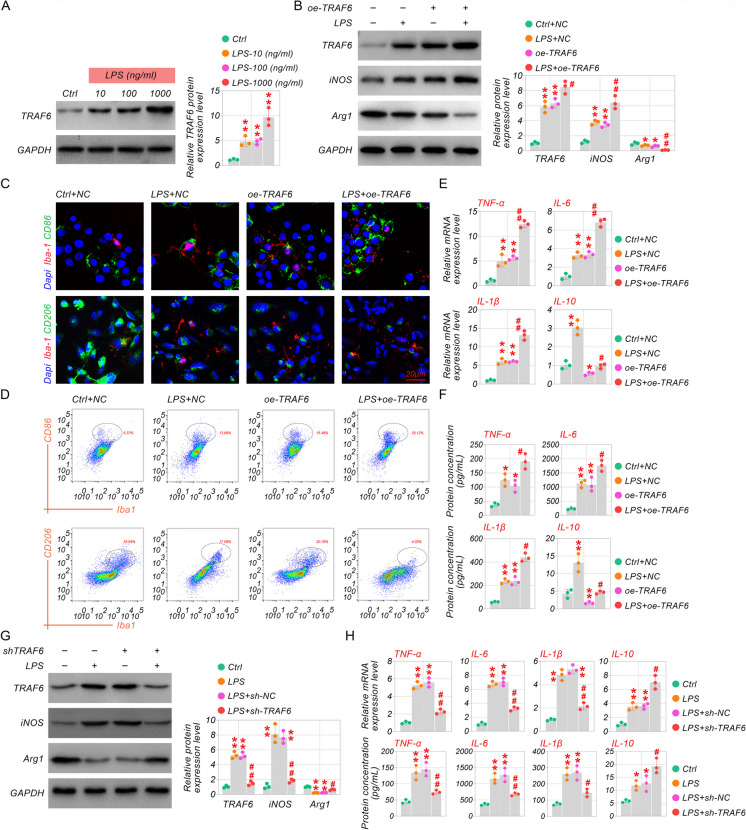
TRAF6 promoted polarization of microglia M1 phenotype. (**A**) Western blot assay was utilized to probe the TRAF6 level in LPS with different doses. (**B**) Western blot assay assessed the TRAF6/iNOS/Arg1 expression in LPS-stimulated BV-2 cells, with or without oe-TRAF6. (**C**) The representative images of immunofluorescence staining of Iba-1 co-expression with CD86 and CD206. (D) Flow Cytometry was applied to quantify the content of CD86 and CD206. (E) Determination of IL-1β/IL-6/TNF-α/IL-10 in spinal cord tissues by qRT-PCR and (F) ELISA. (G) Western blot assay assessed the TRAF6/iNOS/Arg1 expression in LPS-stimulated BV-2 cells, with or without sh-TRAF6. (H) QRT-PCR and ELISA tested the inflammatory cytokines. *p < 0.05, **p < 0.01 vs Ctrl or Ctrl + NC, and ^#^*p* < 0.05, ^##^*p* < 0.01 vs LPS or LPS + NC. Scale bar: 20 μm

To demonstrate the influence of TRAF6 on microglia polarization, we developed a cellular model by stimulating BV-2 cells with LPS, and the exogenous plasmids of TRAF6 were transfected into BV-2 cells. We analyzed the expression of TRAF6, iNOS, and Arg1 using western blot and qRT-PCR, while the proportion of M1 (CD86^+^) and M2 type (CD206^+^) cells detected by immunofluorescence and flow cytometry. As displayed in Fig. 4A, the mRNA and protein expression levels of TRAF6 rose with increasing doses of LPS. LPS-induced enhancement of TRAF6 and iNOS were raised further by overexpression of TRAF6. Inversely, the expression of Arg1 was reduced by exogenous TRAF6 in LPS-exposed BV-2 cells (Fig. 4B). Furthermore, we detected markers of microglia polarization via immunofluorescence co-staining with Iba-1. As shown in Fig. 4C, LPS-stimulated CD86 (M1 marker) and the reduced CD206 (M2 marker) were elevated and inhibited by exogenous TRAF6, respectively. Regarding the flow cytometry results, depletion of TRAF6 decreased CD86, while CD206 conversely (Fig. 4D), the same was true for immunofluorescence. Therefore, we knocked down the expression of TRAF6 in BV-2 cells. Then we determined the level of inflammatory cytokines using qRT-PCR and Elisa and revealed that interference with TRAF6 expression effectively diminished the promoting effect of LPS on pro-inflammatory factors (IL-1β, IL-6, and TNF-α) and up-regulated IL-10 levels (Fig. 4E-F). In addition, we also knocked down TRAF6 in LPS-cultured BV-2 cells to further determine its effect on microglial polarization. It was observed that Knocking down TRAF6 inhibited iNOS expression and promoted Arg1 expression (Fig. 4G). Also, regulating inflammatory factors had the opposite effect on TRAF6 overexpression (Fig. 4H). These results manifest that TRAF6 mobilization of microglia may share the same influences as LPS and that TRAF6 depletion significantly reverses the direction of microglia polarization and the inflammatory state.

### TRAF6 conduces to c-JUN/NF-kB pathway activity in microglia

**Fig. 5:**
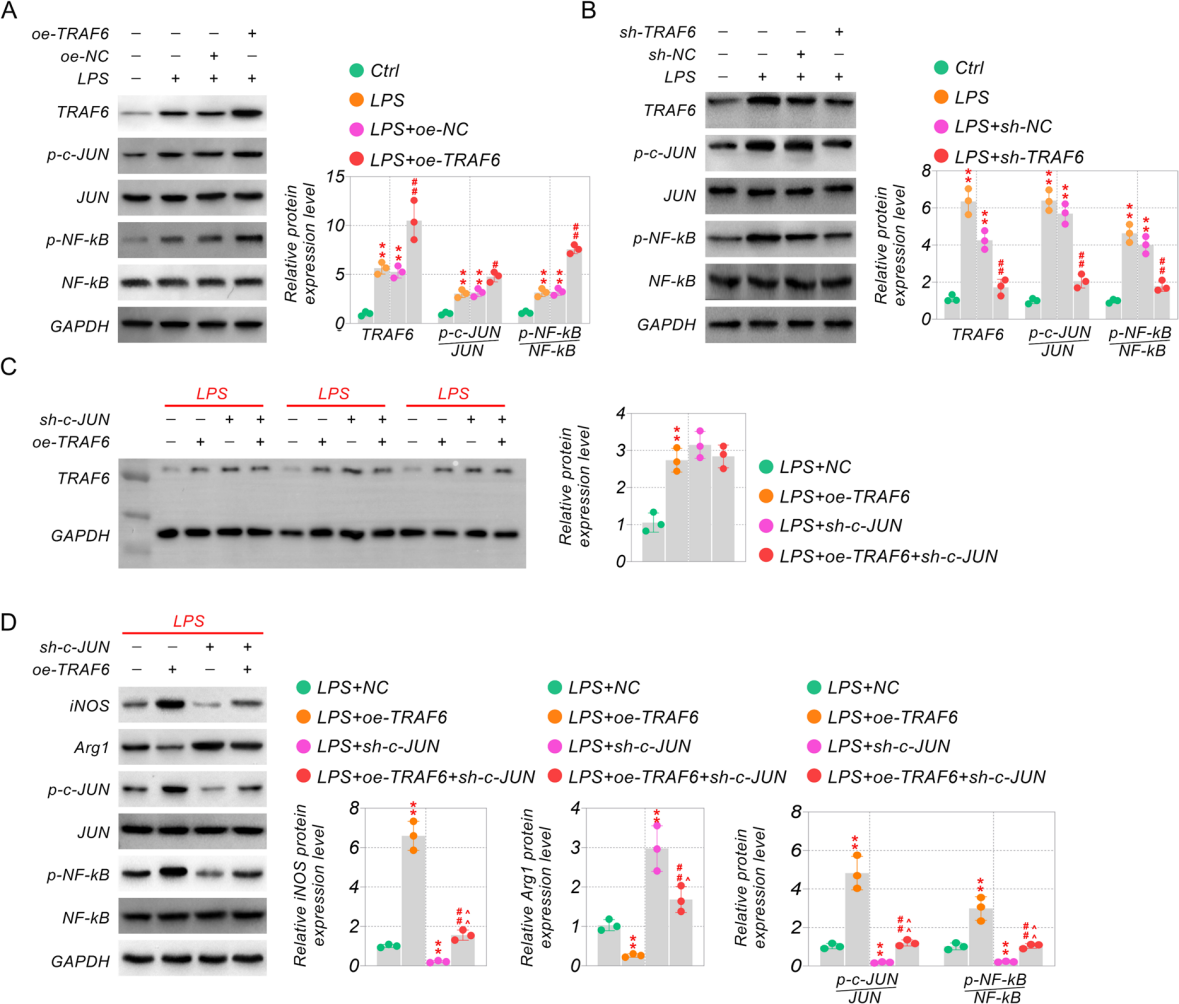
TRAF6 was directed to the c-JUN/NF-kB pathway activity in microglia cells. (**A-B**) Western blot assay assessed the TRAF6/p–c-JUN/c-JUN/p-NF-kB/ NF-kB expression with TRAF6 or sh-TRAF6 in LPS-stimulated BV-2 cells. (**C**) Cells were co-transfected with TRAF6 plasmid and sh-c-JUN, then the proteins of TRAF6, and (**D**) iNOS/Arg1/p–c-JUN/c-JUN/p-NF-kB/NF-kB were determined by WB assay. **p < 0.01 vs Ctrl or Ctrl + NC, ^##^*p* < 0.01 vs LPS or LPS + TRAF6, and ^^^^*p* < 0.01 vs LPS + sh-c-JUN

It had been reported that the effect of TRAF6 on inflammation was concerned with the c-JUN/NF-kB pathway in keratinocytes (Jiang et al. 2022). The inhibition of the c-JUN or NF-kB pathway could reduce M1 polarization of microglia to improve neuroinflammation (Bi et al. 2022; Zheng et al. 2020). To characterize whether the effect of TRAF6 in neuropathic pain involves the c-JUN/NF-kB pathway, we characterized the mobilization of the c-JUN/NF-kB pathway in LPS-stimulated BV-2 cells using western blot. It turned out that the levels of p–c-JUN and p-NF-kB were raised in LPS-stimulated BV-2 cells, and TRAF6 overexpression further stabilized the p–c-JUN and p-NF-kB expression (Fig. 5A). However, the raised levels of p–c-JUN and p-NF-kB were inhibited by silencing TRAF6 in LPS-stimulated BV-2 cells, while the c-JUN and NF-kB expression was not different between these groups (Fig. 5A-B). To further prove the results, we tested the protein levels of the c-JUN/NF-kB pathway under overexpression of TRAF6 and knockdown of c-JUN in LPS-stimulated BV-2 cells.Our data demonstrated that the levels of p–c-JUN and p-NF-kB were raised, and overexpression of TRAF6 further promoted the activity of the c-JUN/NF-kB pathway in LPS-stimulated BV-2 cells (Fig. 5C). Nevertheless, the increased levels of p–c-JUN and p-NF-kB were inhibited by silencing c-JUN co-transfected with TRAF6 plasmid and sh-c-JUN in LPS-stimulated BV-2 cells (Fig. 5C). In addition, iNOS expression was upregulated upon TRAF6 overexpression and downregulated upon c-JUN knockdown, whereas the expression of Arg1 was utterly opposite in LPS-stimulated BV-2 cells (Fig. 5C). These findings demonstrates that TRAF6 modulates microglia polarization status through the c-JUN/NF-kB pathway.

### Inhibition of the c-JUN/NF-kB pathway attenuates CCI-induced neuropathic pain

**Fig. 6:**
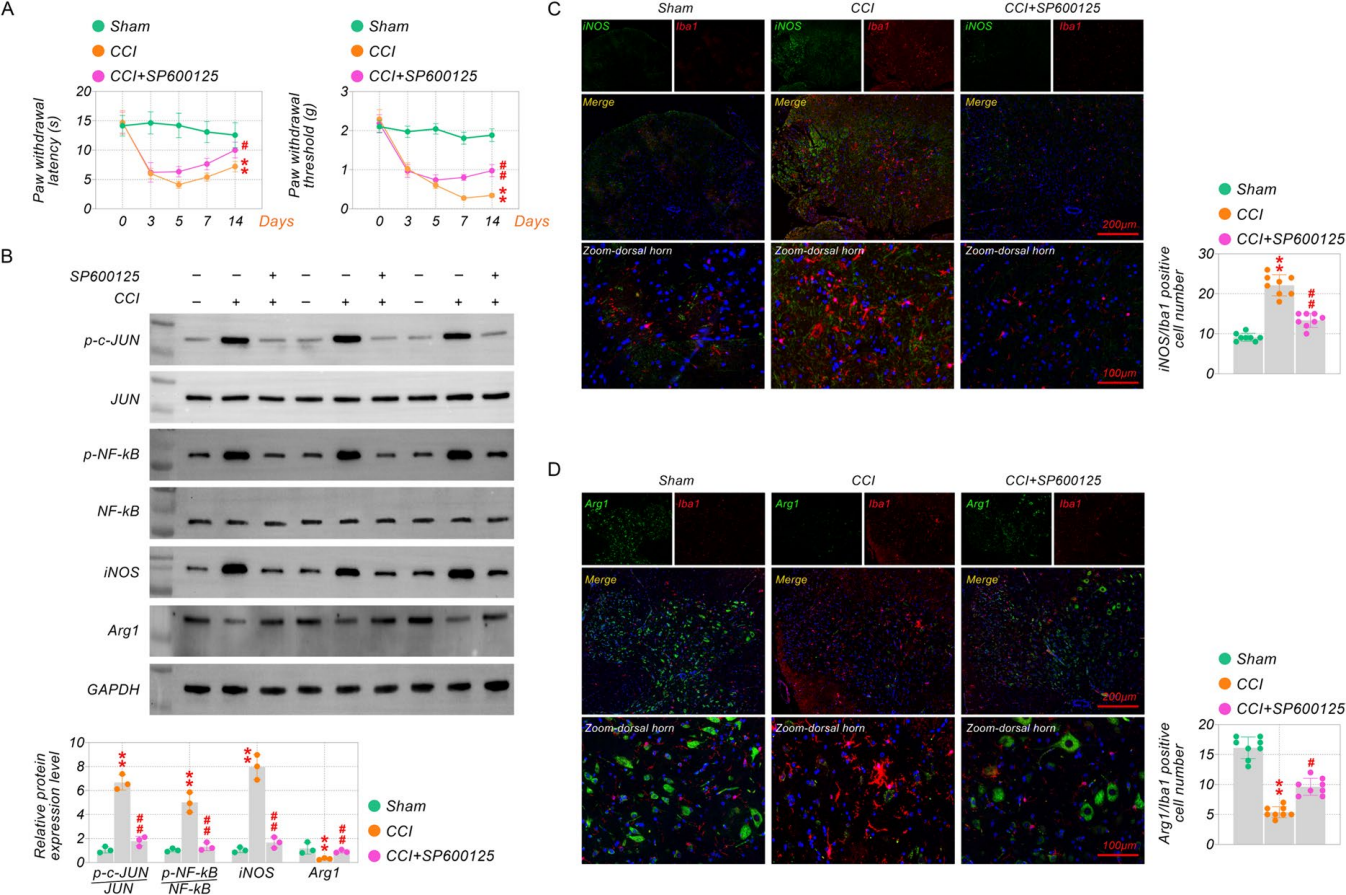
Inhibition of the c-JUN/NF-kB pathway attenuates CCI-induced neuropathic pain (**A**). PWT and. PWL tests. (**B**) The protein levels of iNOS/Arg1/p–c-JUN/c-JUN/p-NF-kB/NF-kB were analyzed by western blot assay in CCI-induced mice treated with SP600125. (**C-D**) The immunofluorescence staining was performed to detect Iba-1, iNOS, and Arg1 levels in CCI-induced mice treated with SP600125. **p < 0.01 vs Sham, and ^#^*p* < 0.05, ^##^*p* < 0.01 vs CCI. Scale bar: 200/100 μm

c-JUN/NF-kB pathway works in neuropathic pain by regulating spinal microglia activation (Tatsumi et al. 2015; Jing et al. 2019). To further explore the effect of the c-JUN/NF-kB pathway on TRAF6-regulated neuropathic pain, c-JUN inhibitor SP600125 was applied in this study. As shown in Fig. 6A, the PWT and PWL were declined by SP600125 treatment from day 5 compared with the CCI group. The p–c-JUN and p-NF-kB were enhanced in the CCI group compared to the Sham group, which was tested by western blot assay. Meanwhile, p–c-JUN, p-NF-kB, and iNOS levels were also restricted to CCI-induced mice treated with SP600125, but the Arg1 level was increased (Fig. 6B). Moreover, the same effect was shown in immunofluorescence images (Fig. 6C-D). SP600125 further suppressed Iba-1 and iNOS expression and decreased Arg1 expression. These results reaffirm the role of the c-JUN/NF-kB pathway activation in CCI neuropathic pain and neuroinflammation, which may also be a critical regulatory mechanism for microglia polarization.

### 3.7 Graphical abstract

This study explores the role of TRAF6 in neuropathic pain. It was determined that TRAF6 mediates the promotion of microglia M1 polarization through activation of the c-JUN/NF-kB pathway. TRAF6 knockdown or an inhibitor of the c-JUN pathway could diminish neuroinflammation and ameliorate neuropathic pain. Such findings potentially represent a new therapeutic approach to alleviate neuropathic pain.

## Discussion

The injury of the peripheral nerve or central nervous system causes neuropathic pain. The enhanced sensitivity to innocuous or noxious stimulation is a significant characteristic of neuropathic pain (Wang et al. 2020; Grace et al. 2014). Microglia are essential in the pathogenesis of neuropathic pain in the spinal cord. Nerve damage leads to a rapid microglial reaction with activation transition of signaling pathways (Wang et al. 2020; Ferraz et al. 2021). Activated microglia polarized into the M1 phenotype or the M2 phenotype (Zhao and Gao 2019). M1 microglia release the pro-inflammatory cytokines (Yao et al. 2014) and increase sensitization of neurons through the interaction of microglia and neurons to contribute to developing neuropathic pain in the spinal cord (Wang et al. 2020; Ferraz et al. 2021). Hence, regulating microglia activation and associated inflammation might be a latent therapeutic strategy for neuropathic pain. In this research, we identified that TRAF6 was a biomarker, and TRAF6 deduction lessened CCI-induced hyperalgesia, microglia activation, and inflammatory reaction in neuropathic pain.

TRAF6 is involved in the modulation of vascular permeability, angiogenesis, and inflammatory signaling in many diseases (Liu et al. 2019). It has been reported that TRAF6 promoted angiogenesis and neurogenesis and inhibited inflammatory response in mice with acute ischemic stroke (Qiu et al. 2021). Oppositely, it has been found in Li’s research that meningitic Escherichia coli significantly induced TRAF6 in human brain microvascular endothelial cells and upregulated TRAF6 led to increased permeability of the blood–brain barrier (BBB) *in vitro* and *in vivo* (Liu et al. 2019). TRAF6 deficiency A only attenuated the progression of atherosclerosis without altering the inflammatory response in adipose tissue (Aryal et al. 2018). However, TRAF6 induced inflammation in human retinal microvascular endothelial cells (Lu et al. 2018) and human alveolar epithelial cells (Guo et al. 2015); loss of TRAF6 effectively relieved inflammation and prevented the development of diabetic retinopathy (Lu et al. 2018) and LPS-induced acute lung injury (Guo et al. 2015). Thus, it could be seen from the previous studies that the function of TRAF6 in inflammation remained obscure, though TRAF6 had been implicated in the inflammatory response in various diseases. In our research, the enrichment of TRAF6 commenced on day 3 and reached peak on day 7. However, TRAF6 expression declined on days 14 and 21, although it remained highly expressed. This could be attributed to the advanced stage of nerve injury with simultaneous neurogenic inflammation and nerve repair at day 14 (Sayo et al. 2019). This finding reaffirms that alterations in the levels of TRAF6 may well direct the neuropathic pain developmental process.

Considering the essential effects of TRAF6 in neuropathic pain, we knocked down TRAF6 in a CCI mouse model. It was revealed that TRAF6 knockdown significantly ameliorated mechanical pain and thermal nociceptive sensitization in CCI mice. Immunofluorescence analysis prompted that TRAF6 was predominantly available and mobilized in microglia and astrocytes, especially in microglial cells, and rarely in neuronal cells. Knockdown of TRAF6 enlarged neuronal cells, while microglial cells were markedly suppressed, and astrocytes did not vary much in size. Noting that TRAF6 may be of extraordinary significance for microglia activation, we further affirmed the role of TRAF6 in regulating the M1/M2 polarization state of microglia in conjunction with *in vitro*. Immunofluorescence and WB results for Iba-1, the M1 marker iNOS, and the M2 marker Arg1 demonstrated that TRAF6 promotes microglia polarization toward the M1 type, and knockdown of TRAF6 reverses this effect. Anti-inflammatory (IL-10) and pro-inflammatory factors (IL-1β, IL-6, TNF-α) also altered with TRAF6. However, we noticed that the anti-inflammatory factor IL-10 appeared to be elevated in neuropathic pain mice compared with normal mice. In contrast, elevated expression of TRAF6 facilitated its reduction, and diminished expression of TRAF6 augmented the level of IL-10. We conjecture that it might be a compensatory protective mechanism of the organism and that microglia polarization is a dynamically balanced process. Anti- and pro-inflammatory factor levels are constantly shifting, and intervention by third-party means accelerates their progression in one direction or another. Of course, the exact cause will be confirmed in future studies.

CCI-induced chronic pain often presents with abnormal neuronal damage in addition to the most common neuroinflammation. Researchers have observed in CCI mice that hyperactivation of inflammatory factors may be a fundamental cause for increasing neuronal apoptosis in the hippocampal region, which alters cognitive function (Zhang et al. 2023). However, it has been proposed that CCI mice have reduced neurons in the spinal dorsal horn, yet statistically, there is no difference (Cheng et al. 2022). This is agreement with our study that CCI mice do not exhibit a significant reduction of neurons in the spinal dorsal horn. Nevertheless, we did not examine the changes of TRAF6 on hippocampal neurons in CCI. In contrast, in our study, TRAF6 expression levels were strongly correlated with microglia activation status. However, the expression variations between them were not exactly in the same time pattern. However, we speculate that the persistent activation of microglia may be associated with the high TRAF6 expression state. Nevertheless, whether the microglia activation initiated the high TRAF6 expression or the high TRAF6 level facilitated the microglia activation, the causal relationship requires detailed confirmation.

This study found that the c-JUN/NF-kB pathway was activated in CCI mice. Knockdown of TRAF6 could inhibit microglia M1 polarization and inflammatory reaction by effectively interrupting the c-JUN/NF-kB signaling pathway. Studies have shown that an activated c-JUN/NF-kB pathway induces microglial M1 polarization and aggravates neuroinflammation in the nervous system (Kong et al. 2020; Barber et al. 2023; Bai et al. 2023). In the present study, treatment with SP600125 effectively relieved hyperalgesia. It weakened microglia M1 activation induced by overexpression of TRAF6, indicating that TRAF6 could promote the development of neuropathic pain via regulating the c-JUN/NF-kB pathway.

In conclusion, the current study revealed that TRAF6 is an essential part of pathological processes and a promising target for neuropathic pain treatment. Inhibition of TRAF6 observably relieved hyperalgesia, alleviated inflammatory reaction, and reduced polarization of microglia M1 phenotype in neuropathic pain. Moreover, regulating the TRAF6-mediated c-JUN/NF-kB pathway in the spinal cord can be a prospective strategy for managing neuropathic pain.

## Supplementary Information

Below is the link to the electronic supplementary material.

ESM 1 (PNG 2.13 MB) Immunofluorescence observation of lentivirus transfection efficiency

High Resolution Image (TIF 7.23 MB)

ESM 2 (DOCX 11.8 KB)

## Data Availability

The authors declare that all data supporting the findings of this study are available within the paper, and any raw data can be obtained from the corresponding author upon request.
