# Supplementary figures and images for: TRAF6 promotes spinal microglial M1 polarization to aggravate neuropathic pain by activating the c-JUN/NF-kB signaling pathway
Source: Cell Biol Toxicol. 2024 Jul 12;40(1):54. doi: 10.1007/s10565-024-09900-6 (PMC11245438; doi:10.1007/s10565-024-09900-6)

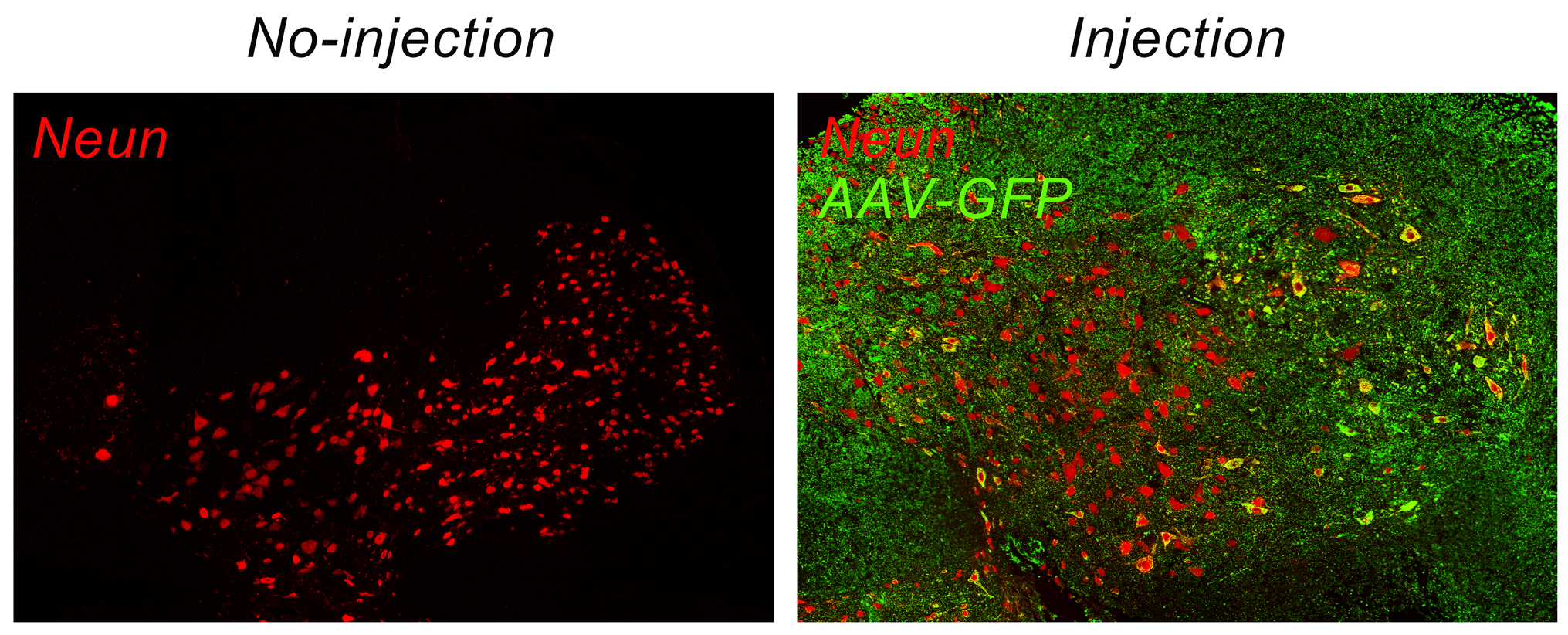

Supplement: Supplementary file 1 — (PNG 2.13 MB) Immunofluorescence observation of lentivirus transfection efficiency [file 10565_2024_9900_Fig7_ESM.png]

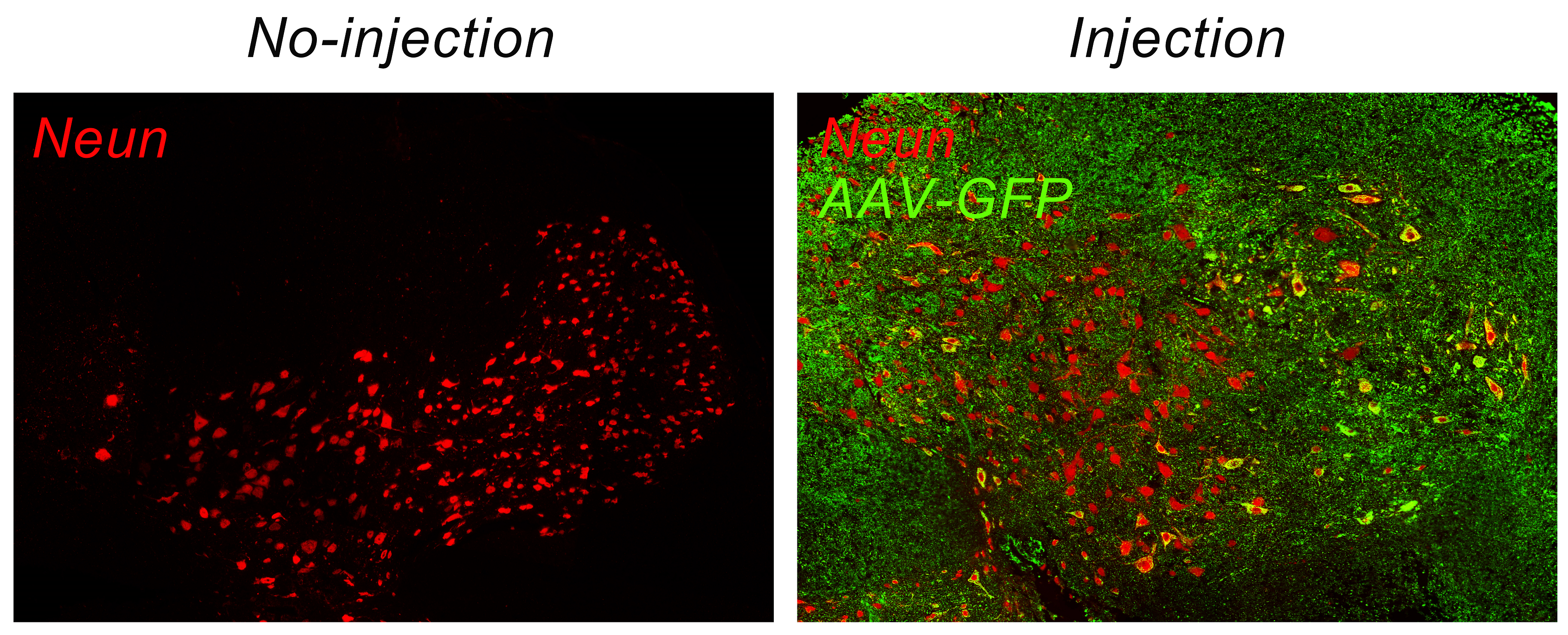

Supplement: Supplementary file 2 — High Resolution Image (TIF 7.23 MB) [file 10565_2024_9900_MOESM1_ESM.tiff]
