# Supplementary material for: TRAF6 promotes spinal microglial M1 polarization to aggravate neuropathic pain by activating the c-JUN/NF-kB signaling pathway
Source: Cell Biol Toxicol. 2024 Jul 12;40(1):54. doi: 10.1007/s10565-024-09900-6 (PMC11245438; doi:10.1007/s10565-024-09900-6)
Supplement: Supplementary file 3 — (DOCX 11.8 KB) [file 10565_2024_9900_MOESM2_ESM.docx]

|  | Forward | Reverse |
| --- | --- | --- |
| TRAF6 | 5’- AAAGCGAGAGATTCTTTCCCTG-3’ | 5’-CAGGCTGTCTTTTGTCAACGA-3’ |
| IL-1β | 5’-AGAGCCCATCCTCTGTGACT-3’ | 5’-CCAGTTGGTAACAATGCCATGT-3’ |
| IL-6 | 5’-GTCCTTCCTACCCCAATTTCCA-3’ | 5’-TAACGCACTAGGTTTGCCGA-3’ |
| TNF-α | 5’-GATCGGTCCCCAAAGGGATG-3’ | 5’-GGTTTGCTACGACGTGGGC-3’ |
| IL-10 | 5’-GGCCCAGAAATCAAGGAGCA-3’ | 5’-CACACTGCAGGTGTTTTAGCTT-3’ |
| iNOS | 5’-CTGCCAGGGTCACAACTTTACA-3’ | 5’-AACAGCTCAGTCCCTTCACC-3’ |
| Arg1 | 5’-AGCCAGGGACTGACTACCTT-3’ | 5’-TTGGGAGGAGAAGGCGTTTG-3’ |
| GAPDH | 5’-GCAGGAGTACGATGAGTCCG-3’ | 5’-ACGCAGCTCAGTAACAGTCC-3’ |

Table S1.qRT-PCR primer sequences

The mRNA levels of TRAF6, IL-1β, IL-6, TNF-α, and IL-10 were determined by SYBR Green (Solarbio, China) and calculated using the △Ct method (2^−△△Ct^) after normalization to GAPDH.
